# Severe Recurrent Necrotizing Myopathy in Pregnancy: A Case Report

**DOI:** 10.3389/fneur.2018.01028

**Published:** 2018-11-28

**Authors:** Yue Li, Lingchao Meng, Yun Yuan, Lijuan Meng, Jing Lin, Bitao Bu

**Affiliations:** ^1^Department of Neurology, Tongji Hospital, Tongji Medical College, Huazhong University of Science and Technology, Wuhan, China; ^2^Department of Neurology, Peking University First Hospital, Beijing, China

**Keywords:** necrotizing autoimmune myopathy, pregnancy, tacrolimus, relapse, immunological evidence

## Abstract

Pregnancy in patients with necrotizing autoimmune myopathy without identified antibodies is rarely reported. We report a case involving a 26-year-old woman with antibody-negative autoimmune necrotizing myopathy who experienced a relapse during pregnancy. Before pregnancy, the patient's myopathy symptoms and elevated serum creatine kinase levels had been successfully controlled with oral prednisone and tacrolimus for 1 year. However, she discontinued the therapy on her own accord, with the aim of conceiving. During pregnancy, she experienced a very severe relapse of muscle weakness and dyspnea and her creatine kinase level increased to >8,000 U/L. After she was treated with intravenous immunoglobulin, oral prednisone, and tacrolimus, she slowly recovered and delivered a healthy neonate. She continues to take oral tacrolimus (3 mg/day) and has remained symptom-free 2 years later.

## Background

Necrotizing autoimmune myopathy (NAM) is a rare muscular disease that is often resistant to conventional corticosteroid monotherapy and needs early aggressive immunosuppressant therapy (1, 2). Unlike the common types of idiopathic inflammatory myopathies (IIMs), such as polymyositis or dermatomyositis, NAM is a less clearly defined category of IIM (3) in which T- and/or B-cell infiltration was scarcely evident. Its occurrence during pregnancy endangers the pregnant women themselves as well as the fetus, which is associated with likely recurrence of symptoms, potential risk of fetal weakness, and potential effects of immunosuppression on pregnancy. Owing to its low incidence (1–9 cases per million per year), only a few studies have described pregnant patients with IIMs (4–12). Pregnant women with active autoimmune diseases pose a potential risk to fertility and disease activity (13, 14). In some studies, IIM is proposed as a risk factor for pregnancy complications. Obstetric complications in patients with IIM included premature birth and stillbirth, but most pregnant patients with IIM reportedly had stable conditions (13, 14). Fertility and pregnancy during the inactive stage of the disease were thought to be much safer than those during the active stage. Obstetricians and neurologists aim at providing therapy based on both disease and pregnancy status, while considering the side-effect of the immunosuppressant. Neck muscle weakness, dyspnea, dysphagia, and heart involvement are common in NAMs (15). Although patients suffer from NAM, its occurrence during pregnancy has not been described thus far.

## Case presentation

A 26-year-old Chinese woman was admitted to Tongji Hospital in 2011 due to the chief complaint of muscle weakness of the upper and lower limbs, which had deteriorated for 6 months. The slowly progressive weakness was associated with slight myalgia. During admission, she was unable to run, climb stairs, or raise her arms over the shoulders. She had no fever, skin rashes, respiratory distress, or dysphagia.

**Figure 1:**
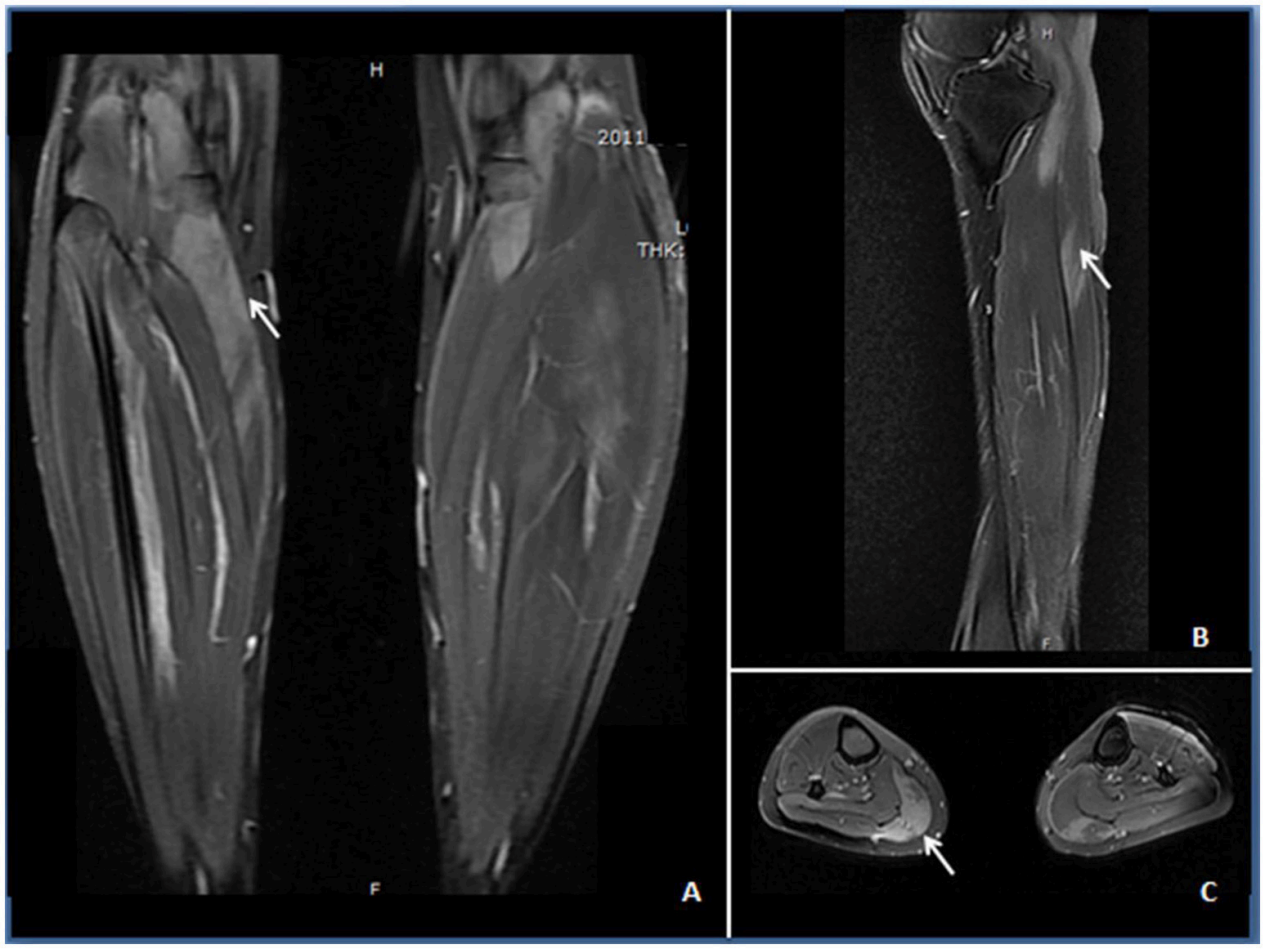
STIR **(A)** and T2-weighted images **(B,C)** demonstrating edema (↑) in the anterior and posterior calves of the patient. **(A)** Increased STIR image signaling in the gastrocnemius with unsymmetrical involvement. **(B)** Increased intramuscular T2 image signaling within the anterior tibial muscle at the sagittal section. **(C)** Patchy T2-weighted hyperintense area in the gastrocnemius, soleus, and anterior tibial muscles.

**Figure 2:**
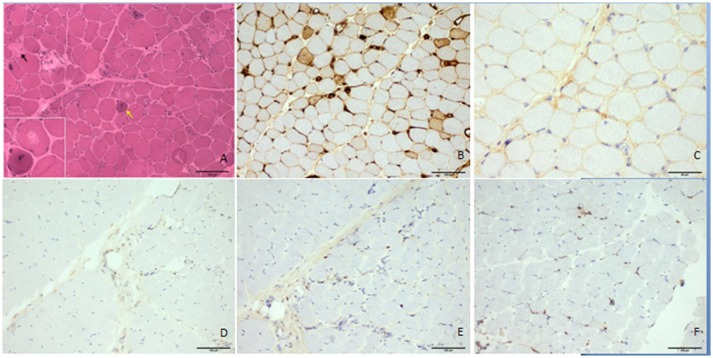
Histologic features of the patient's quadriceps femoris. The hematoxylin and eosin–stained frozen section demonstrated necrotic fibers (▴), regenerating myofibers (↑), atrophic myofibers, and unusual vacuoles in some degenerated myofibers without prominent lymphocytic infiltrates **(A)**. MHC I was upregulated in some myofibers **(B)**, but there was no prominent accumulation of C5b9 in the myofibers **(C)**. CD4, CD8, and CD68 antibodies did not obviously positively stain the infiltrates (**D–F**, respectively). Scale bars: 100 μm **(A–F)** and 50 μm **(C)**.

Neurological examination revealed that her face and neck muscles were not obviously involved. The upper extremities were symmetrically weak (MRC 3/5 on the proximal portion and 4/5 on the distal parts), and the lower extremities were significantly weaker (MRC 2/5 on the proximal portion and 3/5 on the distal muscles). Slight symmetrical atrophy was evident on the proximal weakened muscles, but breathing was not obviously affected. There was no elicited myotonia or skin rash. Laboratory studies revealed increased serum levels of creatine kinase (CK; 12,422 U/L), lactic dehydrogenase (1,156 U/L), glutamic oxaloacetic transaminase (221 U/L), and glutamic-pyruvic transaminase (205 U/L). Tests of antinuclear antibodies, rheumatoid factor, anti-SSA antibodies, paraneoplastic biomarkers, a cohort of specific myositis-associated antibodies (e.g., anti-signal recognition particle antibodies), and *DYSF* gene analysis showed negative results. Electromyographic studies revealed myopathic changes in the proximal muscles. Magnetic resonance imaging revealed the presence of multiple patchy edema in muscles with hyperintense signals in both T2 and STIR imaging (Figure 1). The biopsied muscle from the right quadriceps had widespread myofiber necrosis and regeneration without prominent inflammatory cell infiltration around and within the myofibers (Figure 2). Immunohistochemical staining of the frozen muscle sections for sarcoglycan, dysferlin, and caveolin 3 revealed no deficiency. The library of cluster of differentiation antibodies, such as those against CD4, CD8, and CD68, detected a low number of infiltrates. Major histocompatibility complex (MHC) I was upregulated in some myofibers, but there was no prominent C5b9-positive immunostaining in the myofibers.

Based on clinical grounds, a probable diagnosis of NAM was considered. Initially, the patient was treated with oral prednisone at 40 mg/day. Two months later, the muscle weakness of the limbs did not improve significantly; instead, the clinical status continued to deteriorate with progressive weakness. In 2013, she was hospitalized again. A second muscle biopsy of the left bicep was performed, which revealed changes similar to those in the first biopsy. Intravenous immunoglobulin (IVIGs) at 0.4 g/day for 5 consecutive days was administered. At the same time, oral prednisone at 40 mg/day in combination with oral tacrolimus at 3 mg/day was introduced. Slowly, the muscle strength increased. Six months later, her condition was significantly improved. She was able to raise her arms over her head and stand from squatting position. With the improvement of muscle strength of the limbs, oral prednisone was tapered. One year later, the woman returned to normal life and was able to do office work without any complaints. From then on, she was on oral prednisone at 5 mg/day and oral tacrolimus at 3 mg/day. Her CK level decreased to 385 U/L. She got married and decided to discontinue the therapy to prepare for conception. Although she became pregnant, she experienced severe rebound of muscle weakness including breathlessness during her 6th month of gestation. When the patient came to the Neurology Clinic at Tongji Hospital in 2015, the extremities were very weak (MRC 2/5) and she had difficulty in raising her neck (MRC 2/5). Furthermore, she experienced dyspnea when she moved her extremities. Her CK and myoglobin levels increased to 8,000 U/L and 1,200 ng/L, respectively. Based on the results of fetal monitoring, the patient was hospitalized again and treated with oral methylprednisolone at 30 mg/day, tacrolimus at 1 mg/day, and IVIG therapy at a dose of 0.4 g/kg/day for 5 consecutive days. Mask oxygenation was applied, and vital signs were monitored. The heart rate of the fetus was in the normal range. Again, the patient's muscle weakness slowly improved, and her CK and myoglobulin levels began to decrease gradually. When the patient was due for delivery, she was able to walk slowly without assistance, and had no discomfort or complications resulting from the approximately 2-month pre-delivery treatment with methylprednisolone at 30 mg/day and tacrolimus at 1 mg/day. In April 2016, the patient gave birth through cesarean section to a healthy baby girl with an Apgar score of 10 at 10 min. The infant had a height of 48 cm and a weight of 2,230 g at birth. The tacrolimus and methylprednisolone dose was increased to 3 and 48 mg/day, respectively. The methylprednisolone dose was tapered according to muscle strength and CK and myoglobulin levels. The tacrolimus dose remained unchanged under surveillance of routine blood test and hepatic and renal functions. One year later, she obtained satisfactory clinical recovery. She is currently working in an office again. The serum CK and myoglobin levels were normal on oral tacrolimus 3 mg/day. Methylprednisolone was discontinued. Magnetic resonance imaging of the muscles showed improvement of the edematous change. The baby is currently 2 years old with normal developmental milestones and normal level of creatine kinase.

## Discussion

In this case, the diagnosis of NAM was initially assumed, despite the absence of robust evidence supporting the diagnosis. Later on, the possibility of the diagnosis was further minimized when the first regimen with monocorticotherapy failed to improve the condition. The only clue for possible NAM was the aggressive progression and lack of evidence of limb-girdle muscular dystrophies. Based on the clinical conjecture, a more aggressive therapy was tried with IVIG in combination of oral tacrolimus after the patient signed an informed consent, which significantly improved the symptoms. Based on the effectiveness of the immunotherapy, the diagnosis of NAM was concluded. This implies that there are subcategories of NAMs with yet unknown biologic markers. This case report shows the possible application of trial immunotherapy for myopathies that do not match the diagnosis of muscular dystrophies or other known myopathies.

Unfortunately, our patient experienced a severe rebound of muscle weakness and dyspnea and dramatic elevated levels of CK after she discontinued the drugs and became pregnant. The possible reason for the relapse may be the discontinuation of the medication. In addition, the possible role of the hormones during pregnancy in reactivating the dysimmunity of the mother should not be excluded because some autoimmune diseases become more active during pregnancy (16). According to a report, the activity of some autoimmune diseases, such as multiple sclerosis, neuromyelitis optica spectrum disorders, and rheumatoid arthritis, decreases during pregnancy (17). In China, the recommended treatment is abortion and aggressive medical therapy. However, the patient and her family refused abortion and expressed a very strong wish to keep the fetus. After careful consideration of the potential impacts on the fetus, small doses of methylprednisolone and tacrolimus were administered again in combination with IVIG. The strategy worked well, and the aggressive progression of the disease stopped and reversed, and the woman delivered a healthy girl through cesarean section. It seems that the drugs, hypoxia, and disease did not have any profound impact on the fetus because the baby is growing very well and with normal developmental milestones.

For patients with severe, refractory, or corticosteroid-dependent disease, second-line therapies are frequently used (18, 19). There are many second-line drugs, such as methotrexate, gamma-globulin, azathioprine, cyclosporine, cyclophosphamide, mycophenolate mofetil, and tacrolimus (20–22). However, because of lack of controlled randomized clinical trials, there is no validated therapeutic strategy for NAM (23). In our case, it is regrettable that the woman refused the advice of a neurologist to delay pregnancy or tacrolimus therapy. Given the potential mutagenic effect of immunosuppressants and based on the initial good responsiveness of the patient to tacrolimus, a decreased dose in combination with other immunotherapy was tried to minimize potential harm to the fetus, which was in the 12th week of gestation. Corticosteroids and tacrolimus are two safe medications whose use can be considered throughout pregnancy. Patients are at very low risk of adverse events when corticosteroids are used at a low dose (prednisone < 7.5 mg daily). In contrast, for the rapid control of symptoms, the dose of corticosteroids generally exceed 20 mg/day, which may increase the risk of adverse events, such as preterm delivery and maternal side effects (11, 14, 17). There is little information available regarding the use of tacrolimus during pregnancy, except in cases involving patients with transplants and autoimmune diseases. Fertility impairment due to tacrolimus usage has not been reported (24). The safety of tacrolimus in children was initially observed in our previous study in a cohort of children with ocular myasthenia gravis refractory to corticotherapy (25). However, the use of immunosuppressants in treating pregnant women with autoimmune diseases such as myasthenia gravis, NAMs, and neuromyelitis optica spectrum disorders needs further studies.

This case study highlights the difficulty in recognizing NAM, an increased risk of relapse and complicated pregnancy in patients with IIM, the effectiveness of tacrolimus in the treatment of the disease in combination with other immunotherapy and the possible safety of tacrolimus on the fetus. However, further studies on this condition are needed.

## Ethics statement

Written informed consent was obtained from the patient for the publication of this case report.

## Author contributions

YL, BB, LijM, and JL contributed to the conception and design of the study. LinM and YY performed the immunohistochemical staining. All authors contributed to the manuscript revision and read and approved the submitted version.

### Conflict of interest statement

The authors declare that the research was conducted in the absence of any commercial or financial relationships that could be construed as a potential conflict of interest.
